# Supplementary material for: Design and Synthesis of a Mitochondria-Targeting Radioprotectant for Promoting Skin Wound Healing Combined with Ionizing Radiation Injury
Source: Pharmaceuticals (Basel). 2022 Jun 6;15(6):721. doi: 10.3390/ph15060721 (PMC9229538; doi:10.3390/ph15060721)
Supplement: Supplementary file 1 [file pharmaceuticals-15-00721-s001.zip › pharmaceuticals-1707230-supplementary.pdf]

Supporting Information

Design and synthesis of a mitochondria-targeting radioprotectant for promoting skin wound healing combined with ionizing radiation injury

Zaizhi Du[a,†], Han Liu[b,†], Xie Huang[a], Yang Li[b], Liting Wang[c], Jing Liu[a], Shuang Long[a], Rong Li, Qiang Xiang[b,*], Shenglin Luo[a,*]

[a] State Key Laboratory of Trauma, Burns and Combined Injury, Institute of Combined Injury, Chongqing Engineering Research Center for Nanomedicine, College of Preventive Medicine, Third Military Medical University (Army Medical University), Chongqing 400038, People's Republic of China.

[b] Center of Emergency, First Affiliated Hospital, Third Military Medical University (Army Medical University), Chongqing 400038, People's Republic of China.

[c] Biomedical Analysis Center, Third Military Medical University (Army Medical University), Chongqing 400038, People's Republic of China.

[†] These authors contribute equally to this work.

[*] Corresponding authors: Q. Xiang, xqiang163@163.com; S. Luo, luosl@tmmu.edu.cn

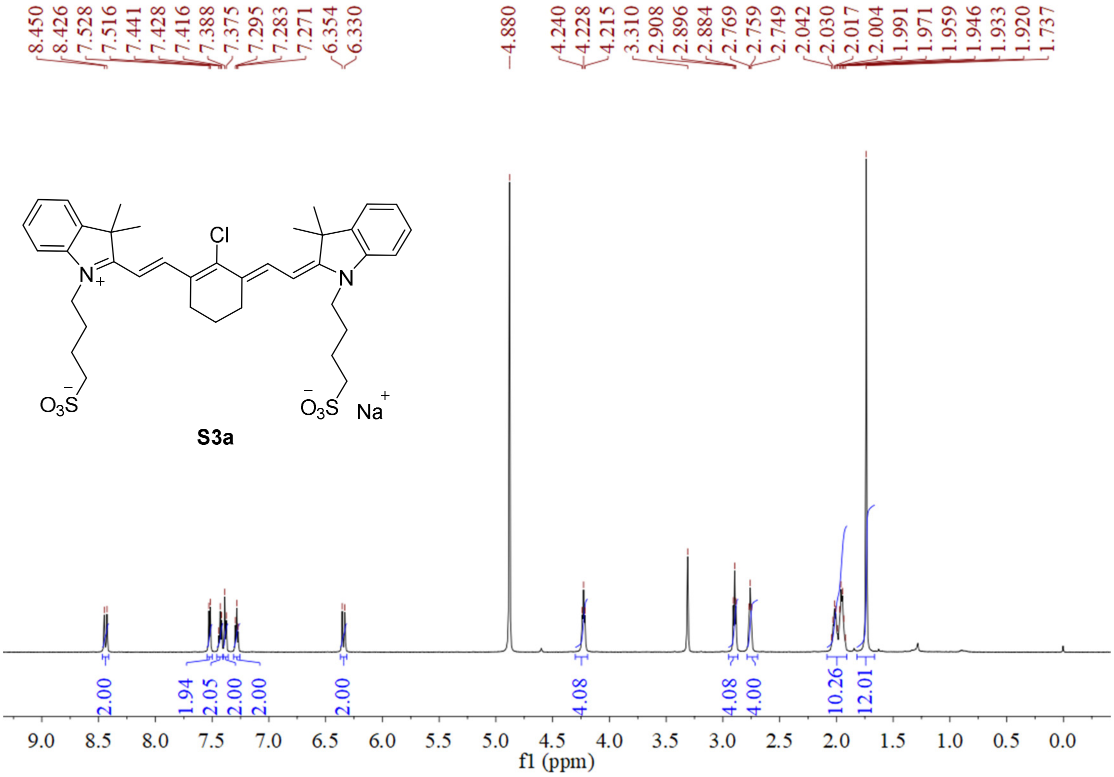

Figure S1. ^{1}H NMR of **S3a**

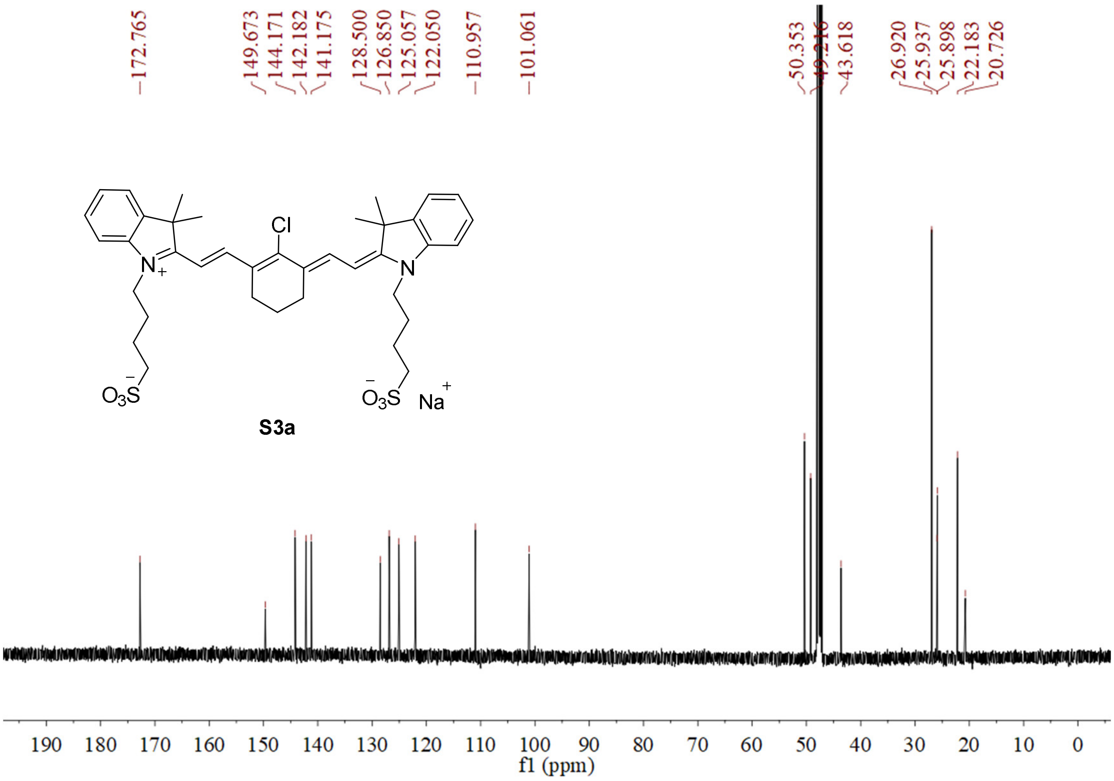

Figure S2. ^{13}C NMR of **S3a**

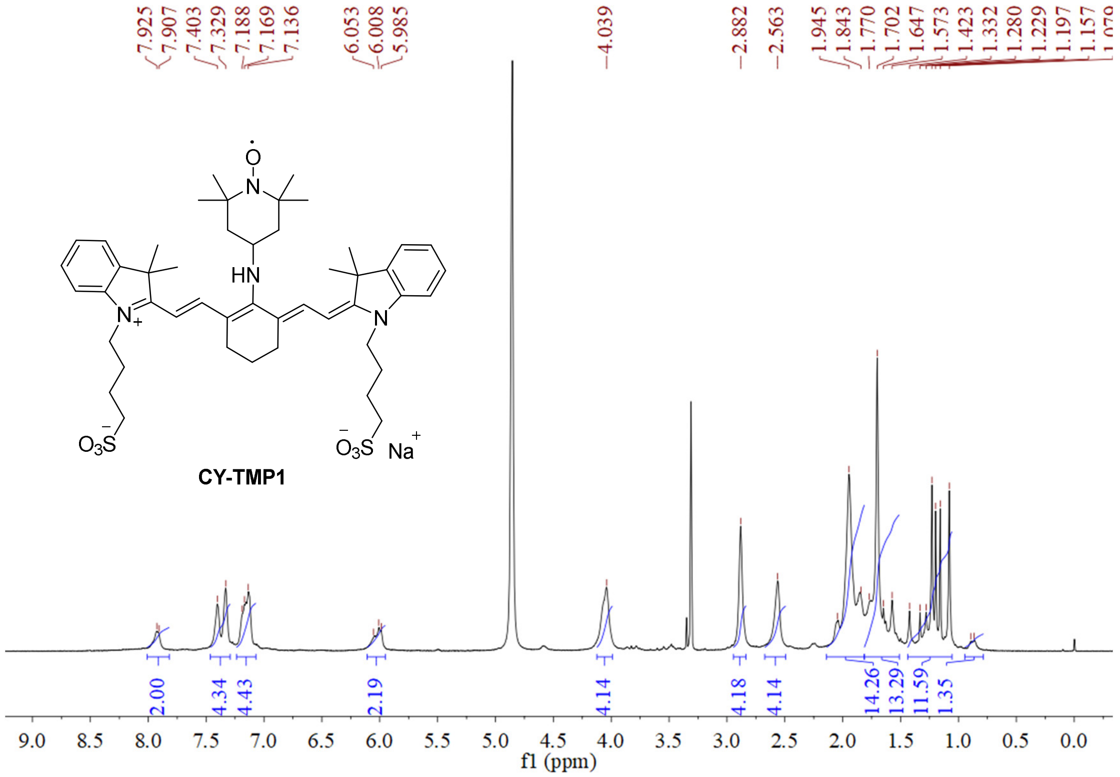

Figure S3. ^{1}H NMR of CY-TMP1

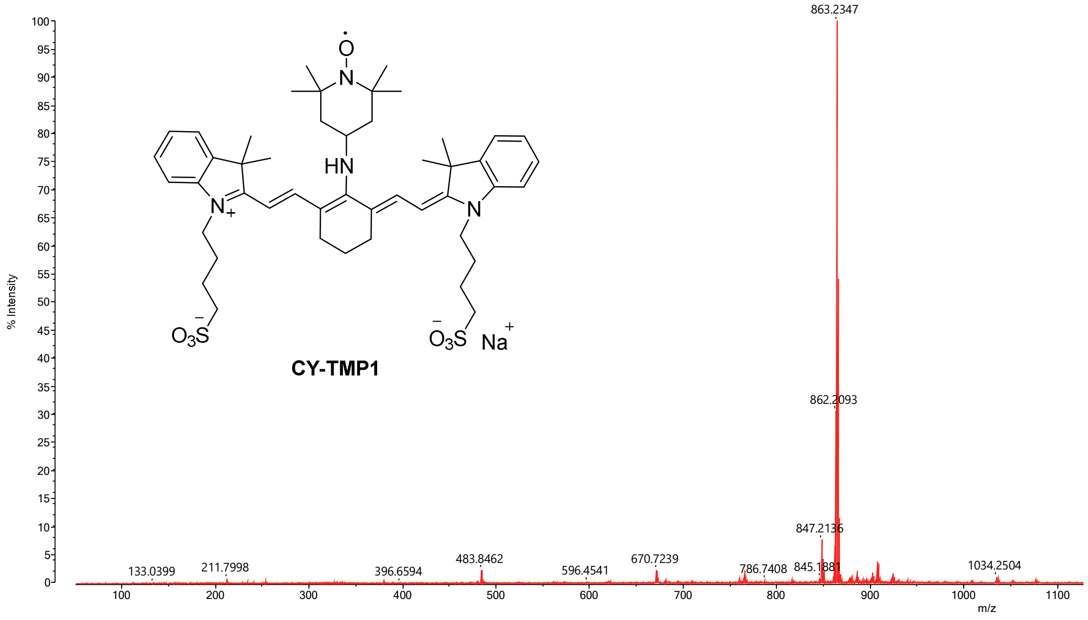

Figure S4. Mass Spectra of CY-TMP1

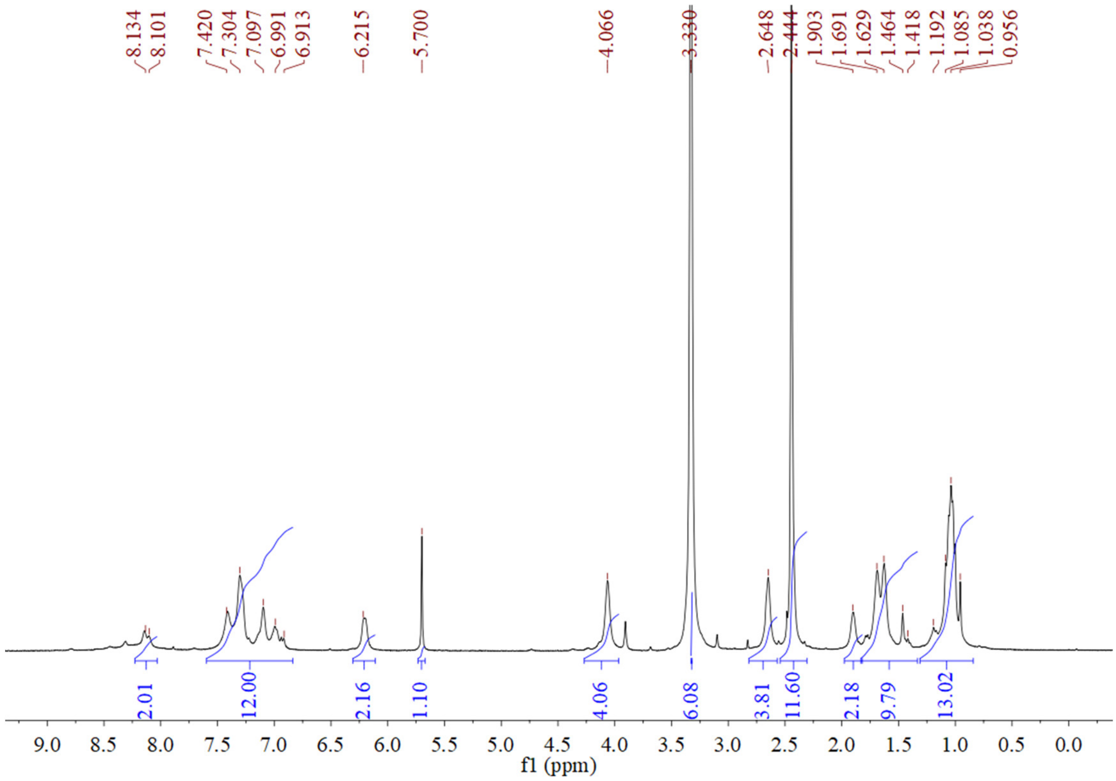

Figure S5. ^{1}H NMR of CY-TMP2

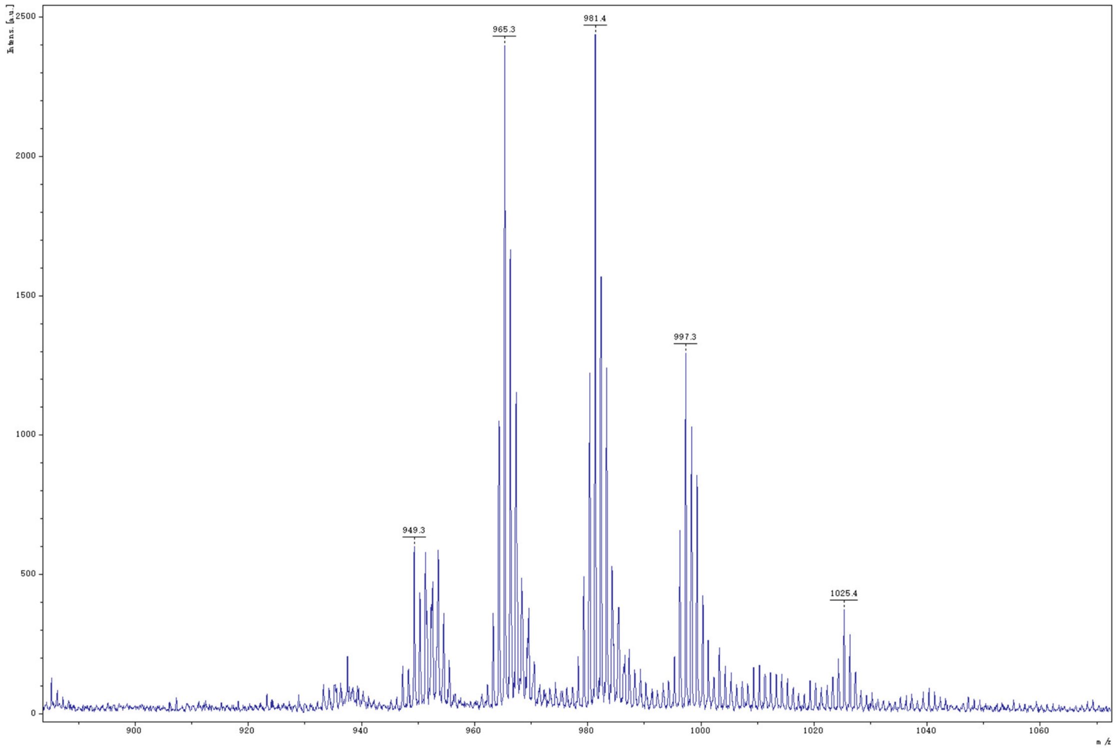

Figure S6. Mass Spectra of CY-TMP2.

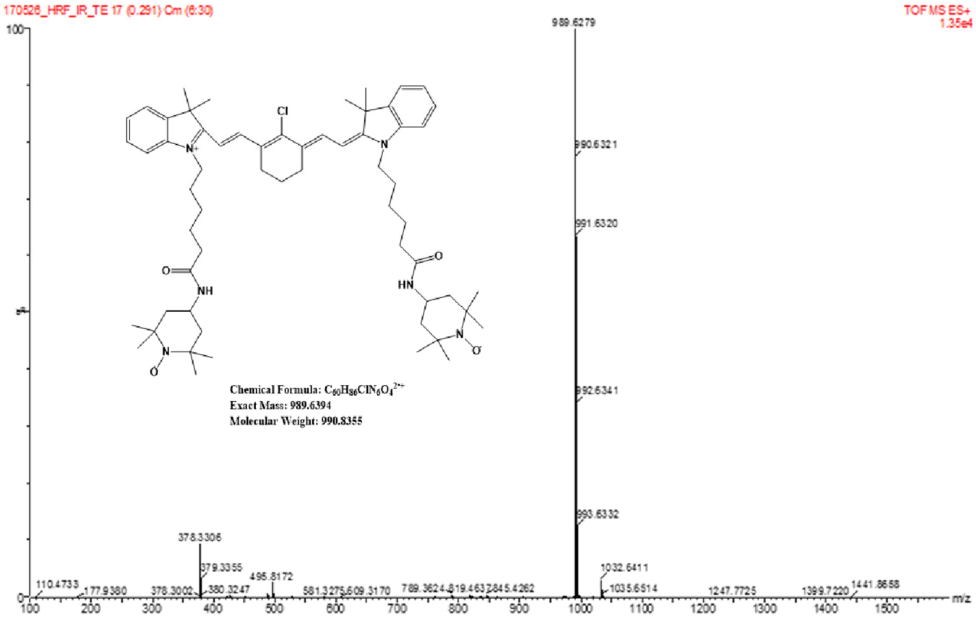

Figure S7. Mass Spectra of CY-TMP3.

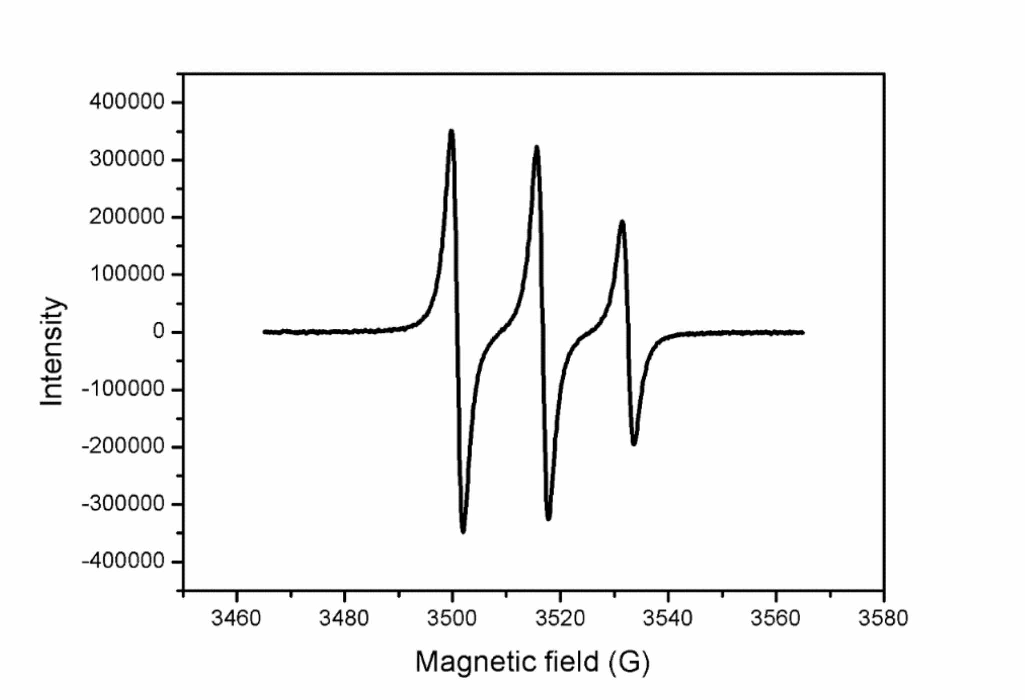

Figure S8. EPR Spectra of CY-TMP1

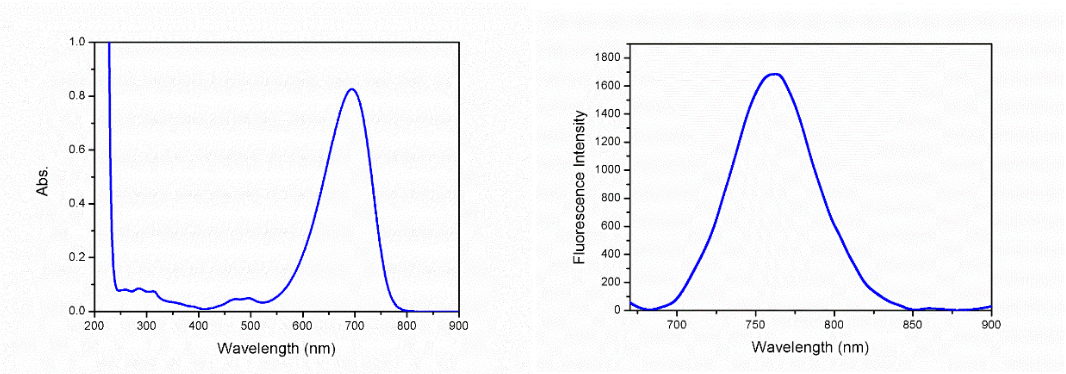

Figure S9. Absorption (left) and emission (right) spectra of CY-TMP1.
